# Transformable Quadruped Wheelchair: Unified Walking and Wheeled Locomotion via Mode-Conditioned Policy Distillation

**DOI:** 10.3390/s26020566

**Published:** 2026-01-14

**Authors:** Atsuki Akamisaka, Katashi Nagao

**Affiliations:** Department of Intelligent Systems, Graduate School of Informatics, Nagoya University, Nagoya 464-8603, Japan; akamisaka.atsuki.d4@s.mail.nagoya-u.ac.jp

**Keywords:** quadruped wheelchair, reinforcement learning, PPO, walking mode, wheeled mode, frequency analysis, distillation

## Abstract

In recent years, while progress has been made in barrier-free design, the complete elimination of physical barriers such as uneven road surfaces and stairs remains difficult, and wheelchair passengers continue to face significant mobility constraints. This study aims to verify the effectiveness of a transformable quadruped wheelchair that can switch between two modes of movement: walking and wheeled travel. Specifically, reinforcement learning using Proximal Policy Optimization (PPO) was used to acquire walking strategies for uneven terrain and wheeled travel strategies for flat terrain. NVIDIA Isaac Sim was used for simulation. To evaluate the stability of both modes, we performed a frequency analysis of the passenger’s acceleration data. As a result, we observed periodic vibrations around 2 Hz in the vertical direction in walking mode, while in wheeled mode, we confirmed extremely small vibrations and stable running. Furthermore, we distilled these two strategies into a single mode-conditional strategy and conducted long-distance running experiments involving mode transformation. The results demonstrated that by adaptively switching between walking and wheeled modes depending on the terrain, mobility efficiency was significantly improved compared to continuous operation in a single mode. This study demonstrates the effectiveness of an approach that involves learning multiple specialized strategies and switching between them as needed to efficiently traverse diverse environments using a transformable robot.

## 1. Introduction

For robots equipped with multiple modes of locomotion, such as walking and wheeled movement, the fundamental challenge lies not in the mechanical design itself, but in the control problem of how to integrate these distinct locomotion strategies and switch between them according to the situation. This research focuses on such locomotion-specific control problems and examines them from the perspective of integrating locomotion strategies. This paper is an extended version of our prior international conference paper [1], and it includes additional experimental results.

### 1.1. Background and Motivation

Despite improvements in accessibility, it remains difficult to completely eliminate obstacles such as uneven road surfaces and stairs, which continue to pose significant mobility challenges for wheelchair passengers [2]. Existing electric wheelchairs may only be capable of ascending or descending standard stairs or straight staircases. Quadruped robots [3,4,5] offer the advantage of being able to navigate diverse terrains, but they face the challenge of low energy efficiency on flat surfaces, as they require a constant power supply from actuators to maintain posture. To address these challenges, hybrid robots combining wheels and legs [6] have emerged, enabling efficient wheel-based movement on flat surfaces and leg-based movement on uneven terrain, thereby enhancing operational efficiency and functional versatility. This study aims to improve the mobility of the transformable quadruped wheelchair [1,7,8] with stair climbing functionality proposed by the authors.

### 1.2. Problem Statement

In order to overcome the mobility challenges faced by wheelchair passengers, it is essential not only to adapt to the surrounding environment but also for the wheelchair itself to have the ability to change its form and drive mode in response to the environment. Conventional stair-climbing wheelchairs only function on well-maintained stairs, and the significant vertical vibrations that occur during ascending and descending are also problematic [9]. Additionally, in hybrid mechanisms with wheels at the feet, continuous torque is required at the elbow joints even during wheeled movement, necessitating the development of a transformation system with high durability to withstand ground impacts and the ability to seamlessly switch between walking mode and wheeled mode.

In this study, we aimed to construct a simulation environment for a transformable quadruped wheelchair and acquire and evaluate control strategies that enable adaptive movement in both modes using reinforcement learning (RL) [10]. Specifically, we quantitatively evaluated the periodic vibrations in each axis direction that occur during walking mode and wheeled mode through frequency analysis, integrated the mode-specific strategies into a single strategy using policy distillation [11], and verified the effectiveness of mode switching through driving experiments on terrain requiring transformation.

### 1.3. Contributions

This paper proposes a new approach to the control and integration of two modes of movement (walking mode and wheeled mode) for a transformable quadruped wheelchair proposed by the authors. First, control strategies for each mode of movement were acquired individually through RL. Next, vibration analysis was performed on both modes, quantitatively demonstrating that the walking mode exhibits greater vertical sway compared to the wheeled mode. This supports the advantage of switching between modes as needed, utilizing the wheeled mode for smooth terrain and the walking mode for rough terrain, based on their respective superior performance in terms of ride comfort and terrain traversal capability.

The innovative contribution of this study lies in the integration of these two separately learned basic posture strategies into a single control strategy using a technique called “mode conditioning distillation.” This integration has enabled the realization of a system in which passengers can actively select their mode of transportation. In simulation experiments, the use of this unified strategy confirmed the possibility of faster movement compared to walking mode alone during long-distance travel.

The true value of unifying strategies lies in their scalability. Unlike methods that switch between separate strategies, unified strategies enable additional learning. This makes it possible to develop more complex and continuous tasks in the future, such as acquiring “transformation actions” when switching modes.

In conclusion, the method of integrating multiple policies by mode conditioning presented in this paper is a versatile fundamental technology that can be applied to all robot systems with diverse motion modes—it is not limited to improving the performance of quadruped wheelchairs.

To promote transparency and reproducibility, we publicly release the full simulation and learning framework used in this study, including the mode-conditioned distillation pipeline, preprocessing/postprocessing modules, and training configurations. The codebase is available at: https://github.com/AkamisakaAtsuki/transformable-quadruped-wheelchair-lab (accessed on 24 December 2025).

## 2. Related Work

### 2.1. Stair-Climbing Wheelchairs

The development of wheelchairs capable of climbing stairs has been attempted for many years, often relying on the crawler mechanism [12]. This mechanism connects multiple wheels with a belt and excels at moving over uneven terrain by increasing the contact area with the ground, but it has difficulty handling complex rotational movements such as those needed for spiral staircases. Additionally, due to the large contact area, the wheels must drag across a wide range of the ground during on-site rotation, leading to reduced stability. As an alternative proposal, there is a planetary wheel system that uses small outer wheels for moving on flat surfaces and large rotating wheels for overcoming obstacles. Quaglia et al. have proposed Wheelchair.q [9], which uses a self-adaptive mobility unit that passively switches between wheeled travel and leg-based walking. However, these systems face significant vertical vibrations when overcoming obstacles, leading to reduced stability on uneven terrain.

### 2.2. Quadruped Robots with Extensions

Quadruped robots have four legs and walk like four-legged animals such as dogs and cats. In recent years, their practical application has been rapidly advancing, especially in complex terrain environments such as construction sites. Quadruped robots demonstrate excellent terrain adaptability, but they have the problem of reduced energy efficiency on flat surfaces. This is because they need a constant supply of power to their actuators in order to maintain their posture. Adding wheels to quadruped robots enables efficient wheeled movement [6]. This hybrid approach optimizes performance on diverse terrains and improves operational efficiency and functional versatility. The concept of transformable robots that can adapt to such environments is attracting increasing attention in robotics research.

Bjelonic et al. [13] proposed an online trajectory optimization framework that solves motion planning for wheeled quadruped robots in real time on board, demonstrating greater robustness against unexpected obstacles. Kashiri et al. [14] developed a robust wheeled legged mobile manipulation platform capable of disaster response and heavy-load logistics tasks that require high loads and aggressive physical interaction. Li et al. [6] presented an extended platform for a high-performance quadruped robot with wheel and leg mode conversion capabilities. This platform is designed to transport cargo in apartment buildings and office buildings and can move at high speeds on flat ground using wheels and move in other areas such as stairs and wheelchair ramps using legs. The wheel arrangement of the transformable quadruped wheelchair proposed by the authors is based on the configuration in Li et al.’s research.

The main difference between the technology in this study and existing approaches is that quadruped wheelchairs are specifically designed to transport people and must constantly support considerable loads. In robots with wheels incorporated into the tips of their legs, the elbow joints must continue to supply torque to support the upper body even in wheeled mode. These joints must also withstand continuous impact from contact with the ground. Therefore, a design that places the wheels directly on the elbow joints may offer higher durability compared to approaches in previous studies. However, implementing such a mechanism would require a conversion system to seamlessly switch between walking mode and wheeled mode.

### 2.3. RL for Locomotion

In recent years, remarkable advances in RL have enabled robots to acquire mobility policies in complex environments [15,16,17]. Here, we overview the main approaches to RL in walking mobility control and clarify the position of the method adopted in this study.

RL is a field of machine learning in which agents repeatedly perform trial and error within an environment to learn policies that maximize cumulative rewards. This process is generally modeled as a Markov decision process [18] consisting of states, actions, and rewards. Approaches to acquiring policies can be broadly categorized into model-based methods [19], which learn the environment model (state transition probabilities), and model-free methods [20], which learn policies and value functions directly without using a model.

In recent years, deep RL, which combines deep neural networks and RL, has become mainstream. In particular, with the advent of DQN [21], which learns value functions, and the Actor-Critic method [22,23], which learns both policy and value functions, applications to robot control with high-dimensional states and continuous action spaces have advanced dramatically. In these methods, numerous studies have been conducted to acquire complex motions by repeatedly performing a large number of trials within a simulator [24].

Learning-based approaches to gait control for quadruped robots can be broadly classified into two types. One is the hybrid approach, which combines a rule-based controller designed by experts with a learning-based method. A representative example of this is PMTG (Policies Modulating Trajectory Generators) [25]. In PMTG, basic leg trajectories (such as Trot and Pace) are first generated using sine waves, and their parameters are fine-tuned to adapt to the environment using RL. While this method has the advantage of stabilizing learning, the generated movements are constrained by the original trajectory generator design, making it difficult to discover completely new movements, and manual design costs still exist.

The other is the end-to-end approach [26,27]. This is a pure RL approach that directly learns from sensor input to actuator command values using a single policy network. Since it does not involve intermediate representations like hybrid approaches, it has fewer artificial constraints and has the potential to achieve more complex, environment-optimized, and effective actions. In recent years, end-to-end approaches have become mainstream due to the discovery of appropriate reward functions.

After considering all of these factors, this study adopted an end-to-end approach. The specific RL algorithm used was Proximal Policy Optimization (PPO) [28], a type of Actor-Critic method that offers an excellent balance between computational efficiency and convergence stability.

Furthermore, although this study is based on simulation, future application to real environments is essential. To this end, techniques such as curriculum learning [29], which gradually increases the difficulty of tasks, and domain randomization [30], which randomizes the physical parameters and appearance of simulators, are known to enhance the generalization performance of strategies learned through simulation and play a crucial role in successfully transferring them to real-world environments (Sim-to-Real) [31,32].

### 2.4. Policy Distillation

Policy distillation [11,33] is a general term for techniques that transfer knowledge from a pre-trained high-performance teacher policy to a student policy. This technique is used not only for the student policy to imitate the teacher policy, but also for integrating multiple teacher policies and generating more practical models. In this section, we first describe the main application objectives of policy distillation, followed by an explanation of its specific technical approaches.

#### 2.4.1. Key Applications of Policy Distillation

Policy distillation is mainly used for the following three purposes.

Model Compression

Large-scale, complex policy networks used in learning offer high performance but come with high computational costs and memory usage. Running them quickly (in real time) on edge devices like robots is not really practical. Therefore, policy distillation is used to compress the behavior of large-scale teacher policies into lighter, more computationally efficient student policies [34,35]. This enables the maintenance of performance while achieving practical inference speeds and resource usage.

Policy Fusion

Multiple specialized strategies tailored to specific tasks (e.g., walking on flat ground, climbing stairs, turning) are also used for the purpose of integrating them into a single general student strategy [36,37,38]. Unlike the method of simply switching between individual policies depending on the situation, integrating them into a single policy is expected to enable smooth coordination between different skills. For example, it becomes easier to acquire complex movements such as “turning while walking.”

Extensibility and Continual Learning

Integrating multiple skills into a single policy greatly improves the scalability of the system. With a group of separate policies, it is difficult to learn additional collaborative actions. However, if they are fused into a single policy through distillation, it becomes possible to add new skills and collaborative actions based on that integrated policy [39,40]. This is an extremely important advantage for robots to acquire new capabilities in the future.

#### 2.4.2. Technical Approaches to Distillation

Distillation from a Single Teacher

The most basic form of distillation is knowledge transfer from a single teacher policy to a student policy. This process involves running the teacher policy in the environment to collect a large dataset of “observations and actions” pairs and then using that data to train the student policy through supervised learning.

The loss function used in this process is selected based on the nature of the teacher policy. If the teacher is deterministic (outputting a single action for a given state), the mean squared error (MSE) is often used. On the other hand, for probabilistic teacher policies like PPO, which output actions as probability distributions, it is desirable to mimic the probability distribution itself. In this case, the KL divergence, which measures the “closeness” of the two probability distributions, is used as the loss function [41]. This makes it possible to transfer not only the results of the actions but also richer information, such as the “confidence level for each action” possessed by the teacher policy, to the student policy.

Policy Fusion from Multiple Teachers

When integrating multiple specialized teacher policies into a single student policy, a more sophisticated approach is required. Simply mixing data collected from each teacher and training the model can lead to issues where behaviors from different tasks interfere with each other, significantly degrading the performance of the student policy [42]. To address this issue, various approaches have been proposed in the context of policy distillation.

In early policy distillation research, attempts were made to separate skills within a single model by switching the output layer (head) of the network according to the task ID [43]. However, in systems such as the transformable robot studied in this research, where physically continuous transitions between modes are required, such a discontinuous network structure is not necessarily optimal.

In recent studies addressing this issue, the mainstream approach has been to integrate multiple skills into a single, fully shared network without separating the output layer. The distillation using mode conditioning adopted in this study belongs to this modern approach. As demonstrated in previous studies such as Actor-Mimic [36], providing the student policy with observation information and mode information indicating “which skill to mimic” enables skill separation within a single unified network. This method is particularly effective for the quadruped wheelchair in this study, which has qualitatively different modes of walking and wheeled movement.

This is because mode information clearly specifies the core action (walking/wheeled movement), while a single network structure provides the basis for smooth transition learning between modes. This is similar to the approach taken by Rudin et al. [40]. This approach differs from those that aim for skill coordination through additional learning in RL without explicit conditioning, such as Rudin et al. [40], as it seeks scalability while ensuring that each skill retains its specialization.

Ultimately, this approach is considered to provide a solid foundation for acquiring the versatility and scalability necessary for student policies to maintain their specialization while also being able to respond to complex tasks through future additional learning.

## 3. Transformable Quadruped Wheelchair

### 3.1. System Overview

**Figure 1:**
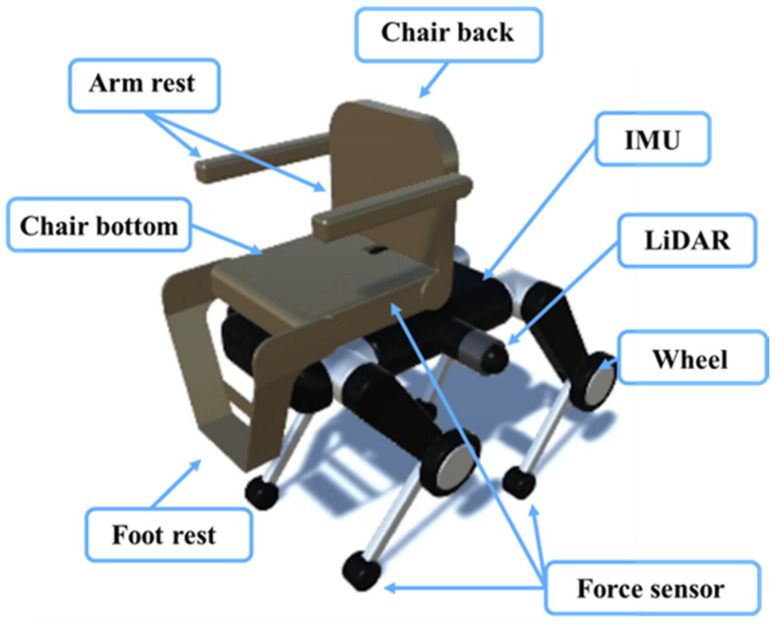
Transformable quadruped wheelchair.

**Figure 2:**
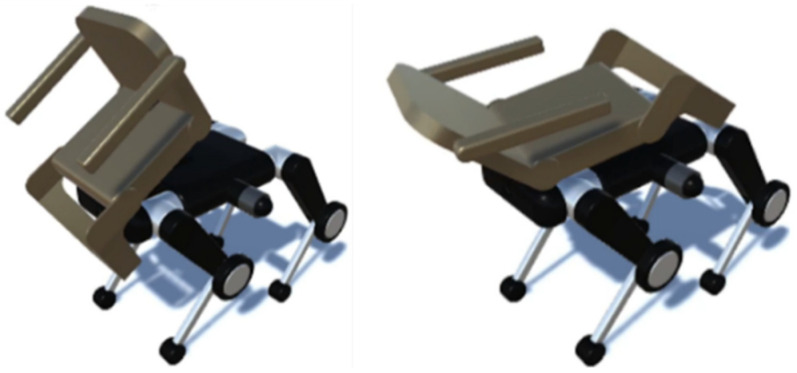
Example of chair transformation.

**Figure 3:**
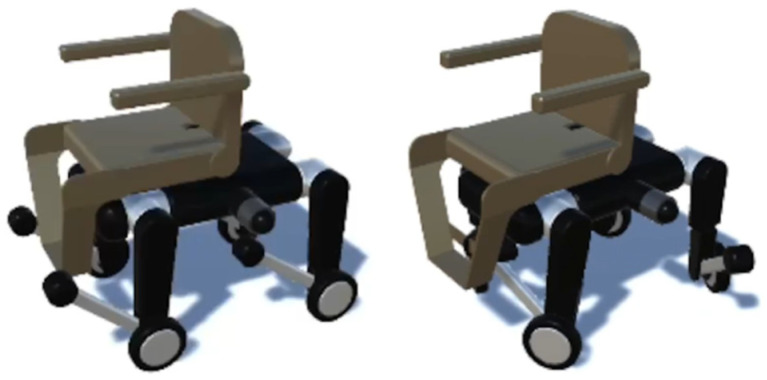
Wheeled transportation. © 2025 IEEE. Adapted with permission from “Acquisition and Evaluation of Walking and Wheel Movement for a Transformable Quadruped Wheelchair Using Reinforcement Learning”, IEEE Proceedings, 2025 [1].

The quadruped wheelchair in this study is shown in Figure 1. It a two-layer structure in which a chair unit with a transformation mechanism is attached to the upper part of a quadruped wheeled robot equipped with wheels at the knee joints. The chair has actuators built into the seat, armrests, and footrests, allowing it to change into multiple forms, such as leaning forward, leaning backward, and rotating, depending on the posture of the passenger and the surrounding environment. Although this functionality was not utilized in the present study, Figure 2 shows forward tilt on the left and backward reclining on the right. This transformation is intended to suppress center of gravity shifts and oscillations that often occur during stair climbing, thereby simultaneously enhancing walking stability and ride comfort. The lower robot uses wheeled movement on flat surfaces and switches to quadrupedal walking on stairs or large obstacles where wheels are ineffective. As shown in Figure 3, by adjusting the wheel orientation through leg rotation, it can perform on-the-spot turns and lateral parallel movement, ensuring high mobility even in narrow indoor corridors or complex outdoor environments.

### 3.2. Hardware Design

#### 3.2.1. Mechanical Structure

**Figure 4:**
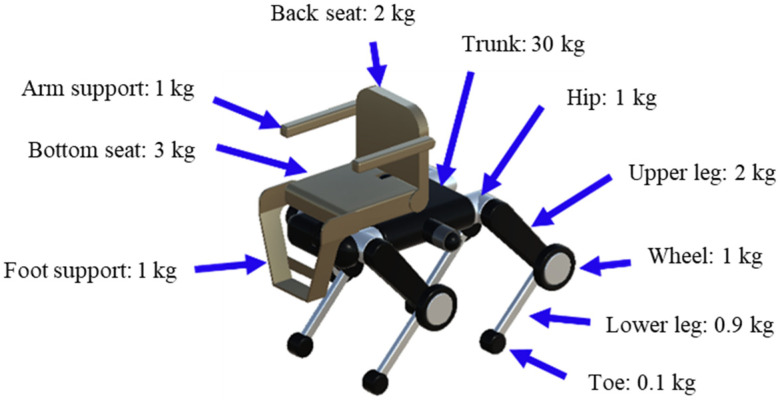
Weight setting of various parts.

**Figure 5:**
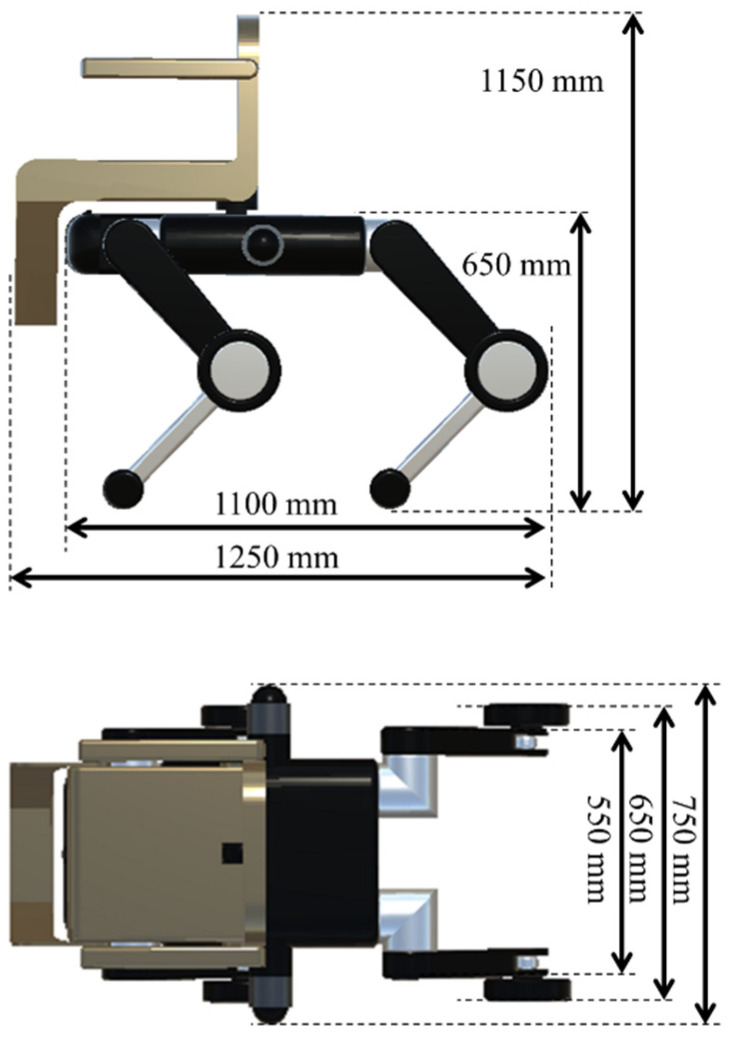
Dimensions of transformable quadruped wheelchair ((**top**) vertical; (**bottom**) horizontal).

**Figure 6:**
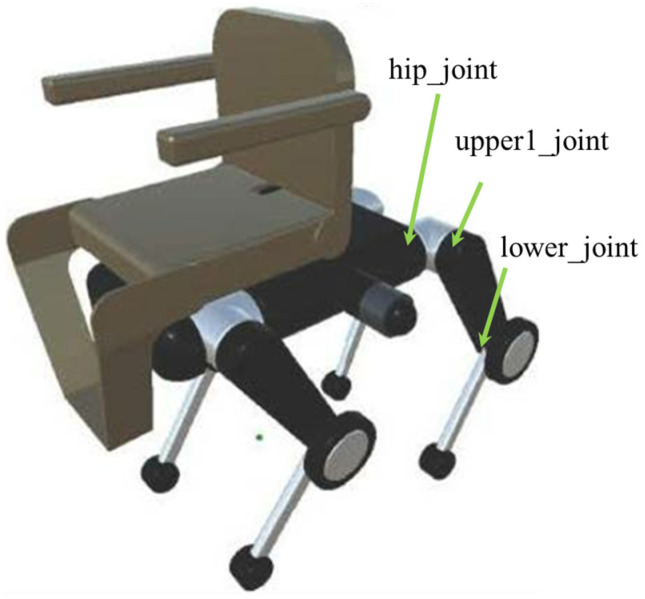
Joints controlled in walking mode.

**Figure 7:**
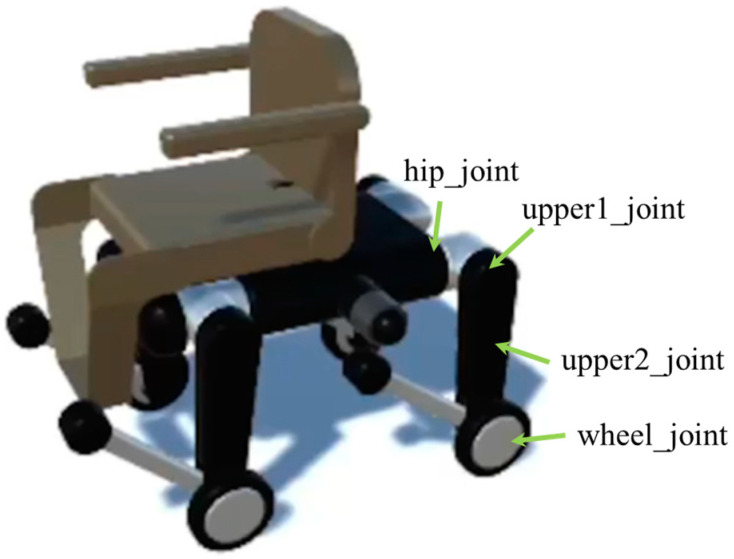
Joints controlled in wheeled mode.

The quadruped robot has a total of 20 degrees of freedom, with each leg consisting of five joints: hip_joint, upper1_joint, upper2_joint, lower_joint, and wheel_joint. The mass parameters are set based on the URDF data [44] of Unitree B2 [4], with the torso weighing 30 kg and each leg having a total weight of 5 kg: 1 kg (hip joint), 2 kg (upper leg), 1 kg (wheel), 0.9 kg (lower leg), and 0.1 kg (toe) from top to bottom. The chair portion consists of a 2-kg backrest, 1-kg arm supports, 3-kg seat, and 1-kg foot supports. Refer to Figure 4 for details on each part and its mass, Figure 5 for the overall dimensions, and Figure 6 and Figure 7 for the joints controlled in the walking and wheeled modes, respectively. The upper2_joint in Figure 7 is an additional rotational joint introduced in this study to enable independent steering of each wheel during wheeled locomotion and does not exist in the standard Unitree B2 configuration.

The overall dimensions are slightly larger than the Unitree B2 due to the addition of the chair and wheels, but the main unit size is nearly identical when the chair is removed.

#### 3.2.2. Actuation and Sensors

Actuator settings are assigned using ArticulationCfg and DCMotorCfg/DelayedPDActuatorCfg in Isaac Lab [45], with appropriate stiffness and response speed profiles assigned to each leg, each wheel, seat, slider, and steering wheel. The leg joints are set for walking, the wheel joints are set for delayed PD control, and the seat area is set for high rigidity, thereby reproducing the behavior of the actual machine in a well-balanced manner.

For environmental recognition, a hemispherical 3D LiDAR [46] on the side of the vehicle body is used to acquire terrain and obstacles, and a 6-axis IMU in the center of the body estimates posture and acceleration. In addition, force sensors at the tips of each leg detect contact force and are used for landing judgment and load distribution. These sensor configurations were selected based on previous research on quadruped robots [47,48,49], and stable walking and fall avoidance are achieved using LiDAR to grasp the terrain, the IMU, and force sensors.

### 3.3. Locomotion Modes

This wheelchair uses RL to learn two strategies: walking mode and wheeled mode. In walking mode, as shown in Figure 6, the three degrees of freedom (hip_joint, upper1_joint, and lower_joint) of each leg (12 degrees of freedom in total) are controlled to move over uneven terrain that is difficult to traverse with wheels, such as stairs and large steps. In wheeled mode, the four degrees of freedom (hip_joint, upper1_joint, upper2_joint, and wheel_joint) of each leg are controlled. In wheeled mode, while independent steering of all four wheels is possible using only the upper2_joint, which handles steering, and the wheel_joint, the risk of falling increases when the rider’s weight shifts or the road surface slopes, causing the robot to lean forward. Therefore, to maintain posture and distribute weight, the hip_joint and upper1_joint are also controlled to allow for fine adjustments to the position of the wheel contact points even while moving.

## 4. RL-Based Locomotion Learning

In this study, in order to acquire policies that can handle both walking mode and wheeled mode, we first adopted a design that allows both modes to be learned independently. This is mainly due to the differences in the reference postures between the two modes. In walking mode, the baseline posture is “standing on four legs”, and it is necessary to acquire movements involving center of gravity shifts and leg lifts. In wheeled mode, the baseline posture is one where the wheels at the elbows or knees are in contact with the ground, and the roles of the joints and the arrangement of support points are fundamentally different. In other words, the two modes are entirely different types of movement that involve mode switching accompanied by transformational movements. Directly applying the same strategy to learn in states with different reference postures can lead to unstable motion acquisition and make it difficult to achieve sufficient performance. Therefore, this study first conducted independent learning for each mode and then aimed to integrate them.

Isaac Lab, which specializes in large-scale parallel learning, was adopted for the learning environment. Isaac Lab can efficiently process thousands of parallel simulations, enabling initial convergence determination, which previously took several days, to be completed in a matter of minutes. This allows for extremely early determination of whether learning is promising, thereby significantly increasing the number of trials for policy design and reward design. However, learning does not complete in just a few minutes; several days of learning are still required for final performance improvement. Nevertheless, the significantly improved feedback speed in the initial stage is highly beneficial from the perspective of research efficiency. In this study, 2048 quadruped wheelchairs were generated in parallel, and learning was conducted while balancing sample efficiency and computational resources.

As the RL algorithm, we adopted PPO. Since PPO is an on-policy method, it can flexibly adapt to curriculum learning that gradually changes terrain difficulty and disturbance conditions during learning. Furthermore, due to its on-policy characteristics, it only uses the latest data from the parallel environment for updates, making it well-suited for large-scale parallel learning.

In this section, we will explain the basic structure common to learning in walking mode and wheeled mode, including an overview of the observation space, action space, and reward design. Detailed specifications and parameter settings for each item will be described next and later.

### 4.1. Policy Network Architecture

Here, we describe the Actor/Critic networks used for RL in walking mode and wheeled mode. Both networks have a multilayer perceptron structure with four fully connected layers. Their layer configurations are shown in Table 1 and Table 2. The input and output dimensions vary depending on the mode, with the two sets of values in the table corresponding to walking mode and wheeled mode, respectively. This is because the number of joints to be controlled differs between modes and the observation vector includes the previous action. To suppress gradient vanishing and stabilize convergence in the early stages of learning, the activation function uses ELUs [50], which maintain a non-zero gradient even in the negative input region. The Critic outputs the value function as a scalar, so the output layer is designed with one unit, while the Actor outputs continuous value actions corresponding to the number of joints to be controlled.

### 4.2. Observation Space

The policy receives a single vector o_t combining each observation group. There are eight observation categories, designed primarily to understand the state of the four-wheeled wheelchair and its surrounding environment. Specifically, the policy uses the following data: body linear velocity (base_lin_vel), angular velocity (base_ang_vel), gravitational direction (projected_gravity), velocity control commands (velocity_commands), joint positions (joint_pos), joint angular velocities (joint_vel), past actions (actions), and surrounding terrain data (height_scan). Each of these items is assigned uniform noise for domain randomization to enhance the robustness of the learned policy. These are basically based on the RL tutorial for quadruped robots published in Isaac Lab [45]. The final observation vector dimensions are 255 in walking mode and 259 in wheeled mode, which are input to each network. Details and dimensions of each observation item are shown in Table 3.

Regarding the differing dimensions of joint_pos and joint_vel in this study, joint_pos is restricted solely to the joints under control. joint_pos represents joint position information, indicating the current angular state of each joint. For controlled joints, explicit target joint angles are provided, and the joint angle itself is directly used as the control variable; thus, its current position holds significant meaning. In contrast, non-controlled joints are fundamentally assumed to remain stationary. Even if they move slightly due to external factors, the resulting position change is negligible. Therefore, we determined that including joint positions in the observations would yield limited information. To reduce the observation dimension, shrink the search space, and enhance learning stability, we excluded joint_pos. On the other hand, joint_vel includes a broader set of joints in the observations, encompassing those outside the control domain. This is because even non-controlled joints can exhibit minute passive motion due to the robot’s movement, contact, or the effects of inertial coupling. Such dynamic changes are easier to capture as velocity information rather than position information.

### 4.3. Action Space

In RL, the action vector $a_{t}$ is a continuous value vector whose dimensions and composition change depending on the mode. In walking mode, it controls the hip_joint, upper1_joint, and lower_joint of each leg, for a total of 12 dimensions. On the other hand, in wheeled mode, hip_joint/upper1_joint continues to be controlled, while lower_joint is removed, and instead, each leg outputs four degrees of freedom (16 dimensions in total) including upper2_joint/wheel_joint. This is because it is necessary to continue controlling joints for posture adjustment in order to prevent forward tilting due to load fluctuations and slopes, in addition to independent steering and driving of the four wheels.

### 4.4. Reward Formulation

The immediate reward $r_{t}$ in this study consists of positive reward terms that encourage target speed tracking, penalty terms that suppress unnecessary behavior, and auxiliary terms that adjust walking-specific properties:

(1) $$\begin{array}{c} r_{t}=1.0\times r_{track\_lin\_vel\_xy\_exp}\\ +0.5\times r_{track\_ang\_vel\_z\_exp}\\ -2.0\times r_{lin\_vel\_z\_l2}\\ -0.05\times r_{ang\_vel\_xy\_l2}\\ -0.00001\times r_{joint\_torques\_l2}\\ -0.00000025\times r_{joint\_acc\_l2}\\ -0.01\times r_{action\_rate\_l2}\\ +0.125\times r_{feet\_air\_time}\\ -1.0\times r_{undesired\_contacts} \end{array}$$

This reward function is based on the RL tutorial for quadruped robots published by Isaac Lab [45]. Details of each remuneration item are summarized in Appendix A.

The first two terms, $r_{track\_lin\_vel\_xy\_exp}$ and $r_{track\_ang\_vel\_z\_exp}$, evaluate the degree of tracking for randomly given planar linear velocity and yaw angular velocity commands using an exponential function, and they are designed so that the reward increases as the robot moves according to the commands. By adopting an exponential function, even small errors are penalized sensitively, and tracking performance improves from the early stages of learning.

The other terms are L2-type penalties designed to suppress unnecessary kinetic energy and sudden changes in behavior. Specifically, they suppress unnecessary translational velocity in the vertical direction (*Z*-axis), unnecessary angular velocity in the plane (XY), excessive joint torque, joint angular acceleration, and the rate of change from the previous action. These are weighted so that they are tolerated within a small error range but are severely penalized for large deviations.

The last two items are auxiliary items specific to the walking mode, where $r_{feet\_air\_time}$ promotes the periodicity of gait by ensuring appropriate airtime for the feet, and $r_{undesired\_contacts}$ severely penalizes unnecessary contact (unexpected contact with the ground or contact at the wrong timing). By combining these, the reward design was created so that a strategy combining tracking, stability, and energy efficiency could be learned.

The reward structure shown in Equation (1) was used for both walking and wheeled modes. However, the two walking-specific items, $r_{feet\_air\_time}$ and $r_{undesired\_contacts}$, were disabled in wheeled mode. This is because foot-ground contact patterns do not exist in wheeled mode. All other items were enabled with the same weighting coefficients to maintain a consistent learning objective across both modes.

### 4.5. Mode-Conditioned Policy Fusion Method

**Figure 8:**
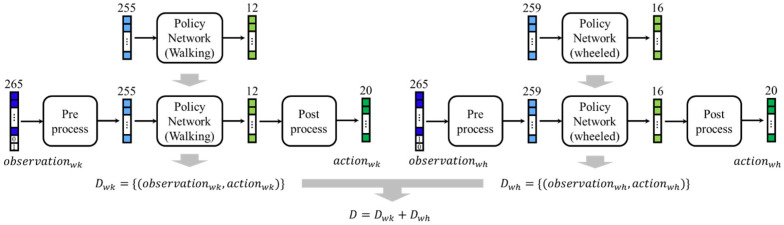
Creation of the mode-conditioned distillation dataset.

To construct a fusion policy conditioned by mode information, it is first necessary to create distillation data conditioned by mode. As shown in Figure 8, there are already independently learned policies for walking mode and wheeled mode, but these were trained in environments where the actuators and offsets of the control target were optimized for each mode during learning. Therefore, to generate distillation data that integrates the two, the forms of observation and action must be matched. Specifically, since there are 20 joints in the four legs excluding the chair, the action vector must also be aligned to 20 dimensions. In walking mode, 12 joints are output from the policy, but the unused joints are fixed at a constant value, which is added to make it 20 dimensions. At the same time, the joint offsets set on the environment side during learning are transferred to the policy side so that the policy output itself becomes the value after the offset is reflected. Similarly, in wheeled mode, the original 16-dimensional output is expanded to 20 dimensions by adding information about joints that were not controlled in wheeled mode, and the offset information is also transferred to the policy side. These are the processes performed in the post-process shown in the figure. On the other hand, since the observations obtained from the environment include 20-dimensional actions with the offsets applied, the pre-process requires the removal of joint information not used during mode-specific policy learning and the subtraction of each offset. Furthermore, one-hot information indicating the desired mode according to the terrain is provided from the environment side, and [0.0, 1.0] is added to the end of the observation when walking mode is requested, and [1.0, 0.0] is added when wheeled mode is requested. By applying the pre-process and post-process in this way, it becomes possible to call and use the policies of both modes, which have been learned individually, within the same environment. Under these settings, the walking mode and wheeled mode are executed separately, and the resulting observation inputs and action outputs are collected. In Figure 8, the data collected in walking mode is denoted as $D_{wk}$, and the data collected in wheeled mode is denoted as $D_{wh}$. The final distillation dataset is created by combining an approximately equal number of each (denoted as D in the figure).

These data are used to perform policy distillation using KL loss. The neural network architecture used for distillation is identical to that employed for learning the walking mode and wheeled mode. The KL divergence is defined as the distance between the probability distributions of the teacher policy $\pi_{T}\left(a|s\right)=N(\mu_{T},\sigma_{T}^{2})$ and the student policy $\pi_{S}\left(a|s\right)=N(\mu_{S},\sigma_{S}^{2})$, as expressed by the following equation.

(2) $$L_{KL}=log\frac{\sigma_{S}}{\sigma_{T}}+\frac{\sigma_{T}^{2}+{\left(\mu_{T}-\mu_{S}\right)}^{2}}{2\sigma_{S}^{2}}-\frac{1}{2}$$

However, since this study deterministically uses only the mean μ of each action during inference, the standard deviation σ was fixed to 1 during distillation. Therefore, the above equation is simplified as follows.

(3) $$L_{KL}=\frac{1}{2}{\left(\mu_{T}-\mu_{S}\right)}^{2}$$

## 5. Simulation Environment

### 5.1. Baseline Terrains and Parallel Training Setup

**Figure 9:**
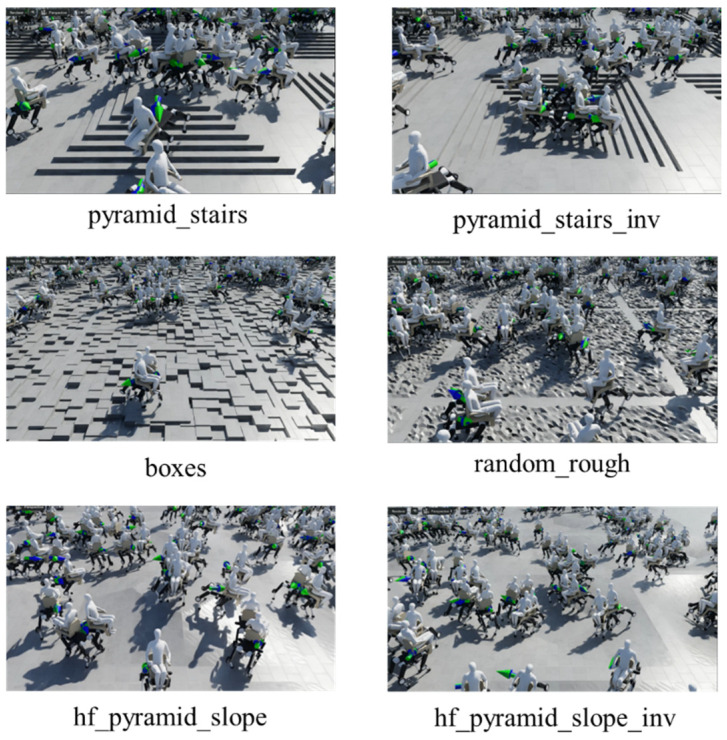
Example of the simulation environment with six types of terrains running in parallel. © 2025 IEEE. Reprinted with permission from “Acquisition and Evaluation of Walking and Wheel Movement for a Transformable Quadruped Wheelchair Using Reinforcement Learning”, IEEE Proceedings, 2025 [1].

**Figure 10:**
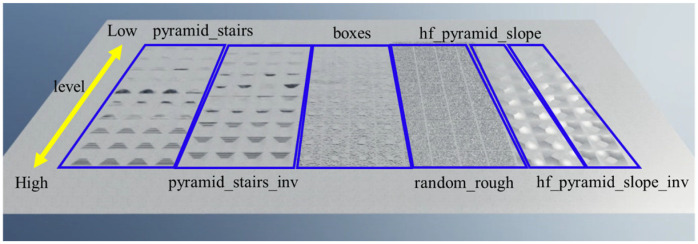
Learning environment in walking mode.

**Figure 11:**
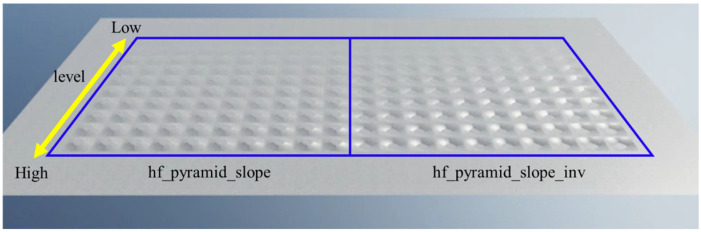
Learning environment in wheeled mode.

**Figure 12:**
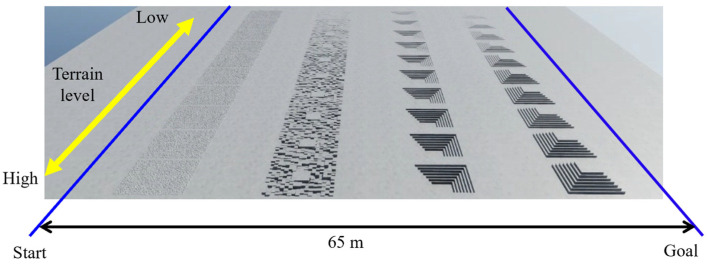
Evaluation environment for policy fusion.

The simulation was conducted on Isaac Sim [51], which generated 2048 quadruped wheelchairs simultaneously for parallel learning. Six types of basic environments were prepared, as shown in Figure 9: pyramid_stairs, pyramid_stairs_inv, boxes, random_rough, hf_pyramid_slope_inv, and hf_pyramid_slope. All of these were used for training in walking mode (Figure 10). On the other hand, wheeled mode learning was limited to the two types of terrain where wheel driving is possible, hf_pyramid_slope_inv and hf_pyramid_slope, and training was continued until the reward converged (Figure 11). For the collection and analysis of vibrations, both walking mode and wheeled mode were executed on the same two types of terrain, and vibration information obtained from acceleration was acquired and analyzed. For the evaluation of the fusion policy, a dedicated course with a maximum travel distance of 65 m was constructed, assuming long-distance travel, and the terrain was arranged so that wheeled mode and walking mode appeared alternately (Figure 12). Each terrain area has predefined attributes such as “Recommended Walking Mode” or “Recommended Wheeled Mode.” We configured the script to trigger a transformation signal when the robot approaches an area boundary. Mode switching is not automatically determined; instead, it is manually controlled based on predefined area information established during simulation. Furthermore, we carefully adjusted the boundary positions and switching timing to prevent unnatural behaviors, such as falling from a step immediately after transformation. In addition, in order to achieve efficient and stable policy acquisition, a curriculum-based learning system with gradually increasing difficulty levels was introduced, starting with easy sections with low kick-up in the early stages of learning and gradually moving to difficult sections with high kick-up as progress was made.

### 5.2. Domain Randomization and Passenger Modeling

**Figure 13:**
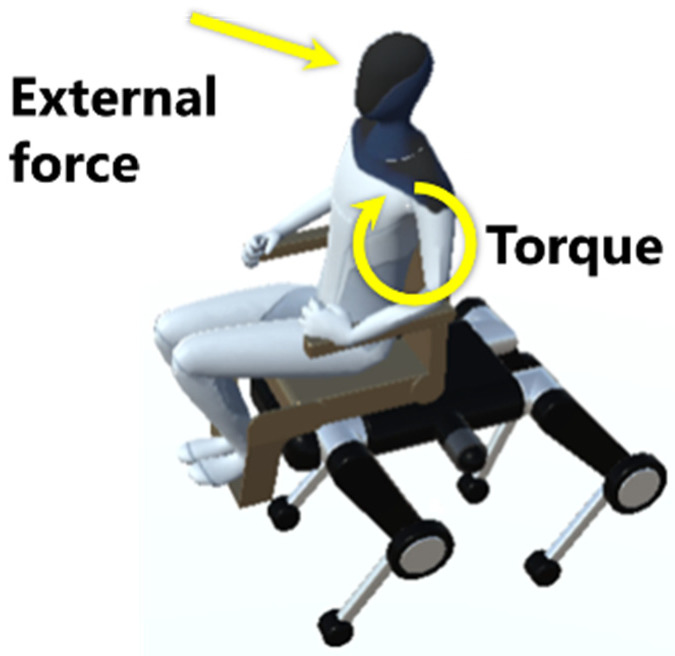
Adding passenger and external disturbances.

As part of domain randomization, the passenger model used the 3D model of Tesla Bot [52], with weight randomly set within the range of 30–60 kg. This range was determined considering the physical characteristics of the intended passengers, as weight changes affect the center of gravity and moment of inertia, making it important to expose the model to a wide range of conditions from the learning stage. To simulate unexpected active and passive movements caused by actual passengers’ random movements, external forces of −10 to 10 N and external torque disturbances of −10 to 10 N·m were applied to arbitrary parts of the passenger during training, as illustrated in Figure 13. The direction of action was randomized to include front-back, left-right, and up-down, and by reproducing various disturbance scenarios, the goal was to acquire robust strategies that can operate stably even under disturbances that may occur in real environments, such as contact impacts and changes in the posture of passengers.

Furthermore, this study explicitly limits the scope of evaluation, recognizing that disturbance magnitude strongly depends on passenger behavior. Specifically, it assumes normal usage conditions where passengers are relatively stable and seated, restrained by seat belts, etc., and targets routine, small-scale disturbances such as breathing, minor posture adjustments, and weight shifts. Under this premise, while the disturbance forces and torques set in this experiment are small compared to the passenger’s body weight, they are considered within a reasonable range for conditions not involving intentional body movements or collisions.

### 5.3. Velocity Command Provision Strategy

The method of giving movement control commands varies depending on the learning objective. In individually learned walking and wheeled mode policies, it is necessary to faithfully follow the speed commands presented randomly within the episode length range. On the other hand, in the evaluation environment for fusion policies, the objective is to reach the goal set at the right end from the starting point, and by adjusting the commands as appropriate during the process, the execution performance for long-distance tasks involving mode switching is verified.

## 6. Experimental Results

### 6.1. Walking Mode Performance

**Figure 14:**
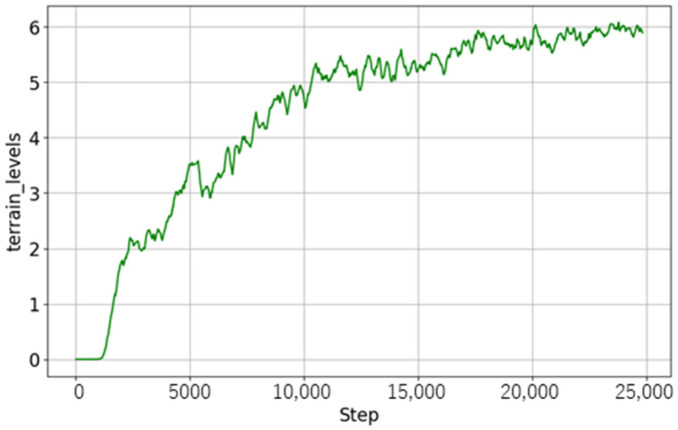
Curriculum progress in walking mode learning.

**Figure 15:**
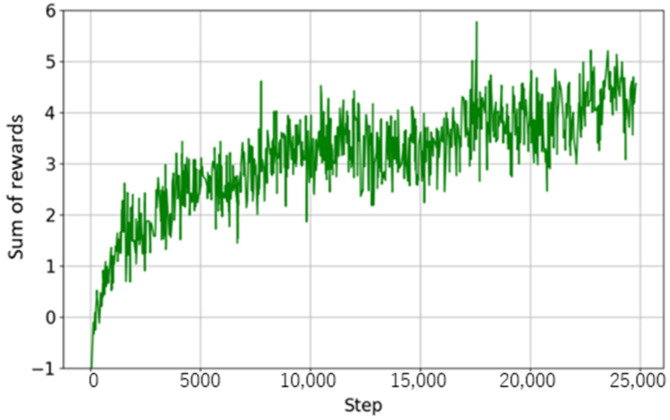
Reward progress in walking mode learning.

Walking mode learning continued until the reward converged through curriculum learning. Learning stabilized at approximately 25,000 steps, and the final reward value also showed a convergence trend. Figure 14 shows the change in curriculum values, and Figure 15 shows the change in rewards. From both figures, it can be confirmed that both the curriculum values and reward values increased as learning progressed and reached a certain value.

The final curriculum value of 6 reached is nearly the upper limit of the difficulty level set in this study, indicating that traversing high-difficulty terrain with steep slopes and uneven surfaces is now possible. From this, it can be concluded that the walking mode policy has achieved stable performance in both fall avoidance and terrain adaptation.

### 6.2. Wheeled Mode Performance

**Figure 16:**
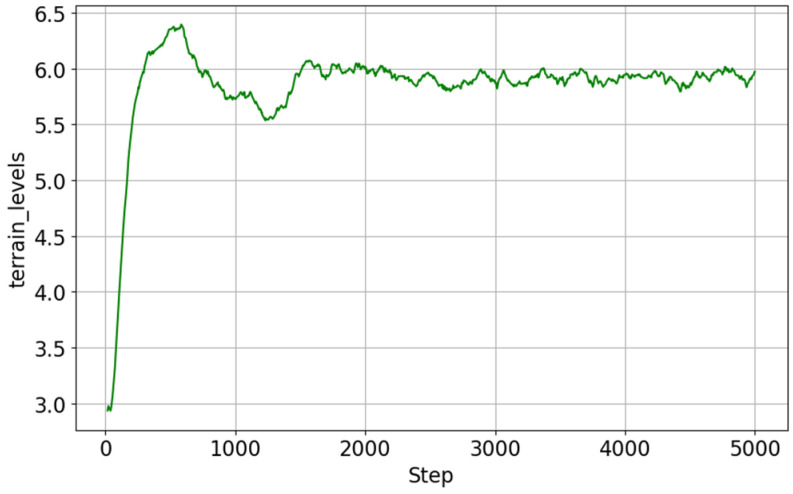
Curriculum progress in wheeled mode learning.

**Figure 17:**
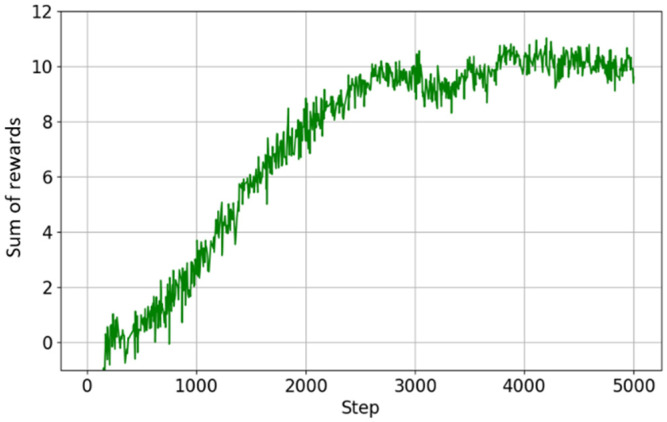
Reward progress in wheeled mode learning.

The learning results for wheeled mode are shown in Figure 16 (curriculum progress) and Figure 17 (reward progress). Learning converged in a relatively short period of approximately 5000 steps, and the reward value reached a higher level than that of walking mode.

This is thought to be largely due to the fact that wheeled mode excludes the high-difficulty elements of walking mode, such as complex steps and uneven ground, and focuses on relatively flat terrain for learning. In addition, wheel-based movement has a lower risk of falls than walking and is easier for long-distance travel, so there is less reward loss during continuous travel. As a result, the curriculum reached its maximum value in a short period of time, and the reward value remained stable at a high level.

### 6.3. Sway Analysis

**Figure 18:**
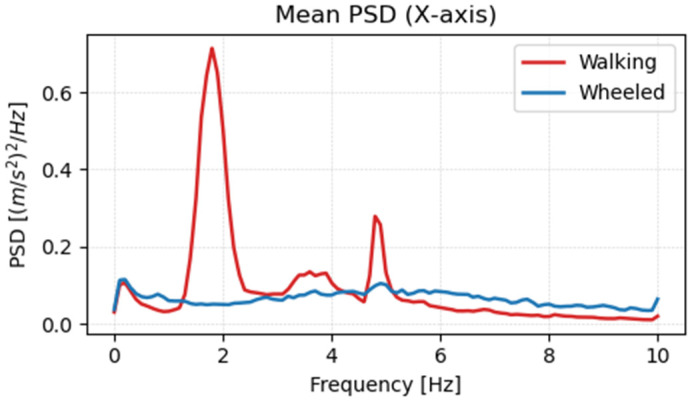
Frequency analysis results of acceleration in the *x*-axis direction.

**Figure 19:**
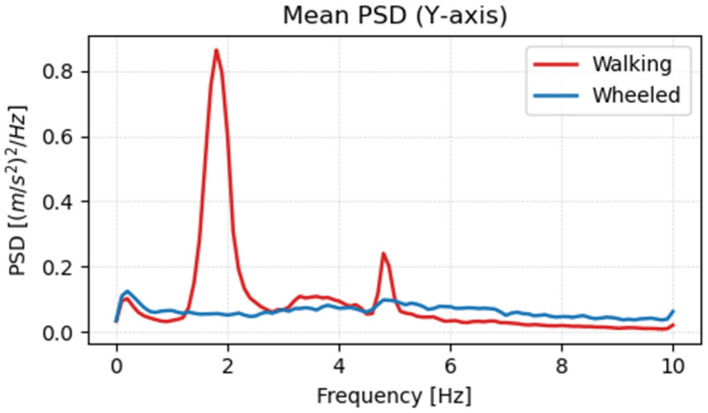
Frequency analysis results of acceleration in the *y*-axis direction.

**Figure 20:**
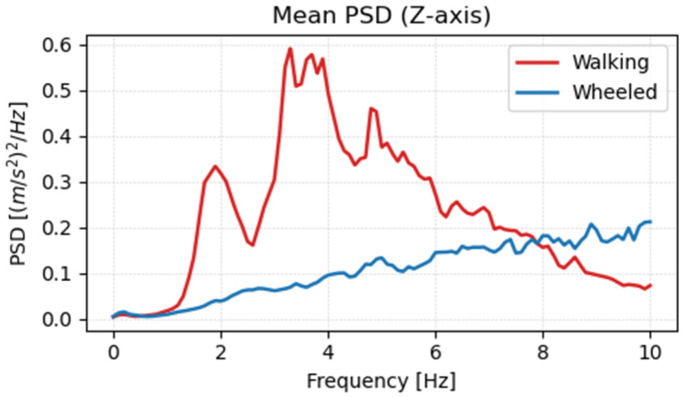
Frequency analysis results of acceleration in the *z*-axis direction.

Using the learned policy, acceleration data were acquired for wheeled mode terrain. Figure 18, Figure 19 and Figure 20 show the frequency analysis results for the *x*-axis, *y*-axis, and *z*-axis, respectively. In walking mode, large vibration peaks were observed around 2 Hz on both the x- and y-axes, and a broad-band vibration component with a peak around 4 Hz was observed on the *z*-axis. In contrast, in wheeled mode, no clear peaks were observed on any axis, and it was confirmed that vibration components were reduced across the entire frequency range. This is believed to be due to the stability of the contact surface provided by the wheels and the continuous rotational motion, which eliminates the leg-lifting actions and landing impacts characteristic of walking.

### 6.4. Policy Fusion Evaluation

**Figure 21:**
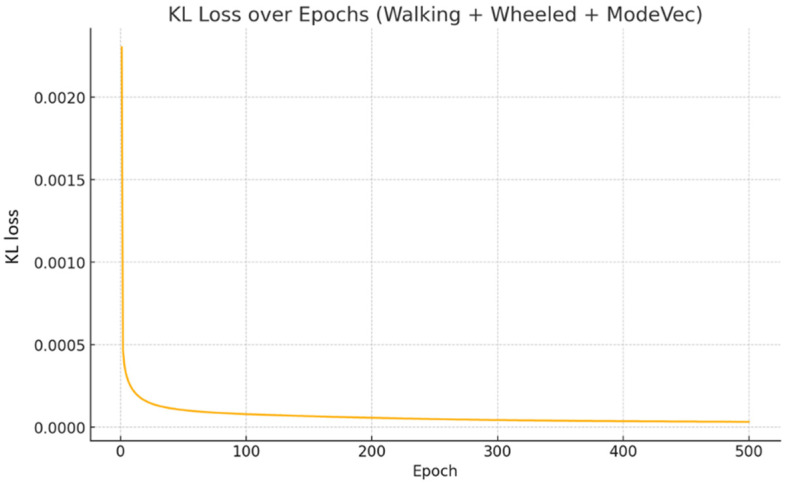
KL loss trends.

We used a dataset generated by assigning mode vectors to observations and actions in walking mode and wheeled mode and performed policy distillation into a single network using KL loss. The number of observations and actions collected here was 1.4 million each for walking mode and wheeled mode, for a total of 2.8 million. Figure 21 shows the change in KL loss when learning using these data. Since convergence was confirmed early on, training was terminated after 500 epochs.

Table 4 compares reward sums of the individual policy and the reward sum of the distilled fusion policy. During inference, the mode of the probability distribution (deterministic action) was adopted, resulting in a higher value than the reward value during learning, which includes exploration. The rewards for the walking mode are 17.51 for the individual policy and 16.87 for the distilled model, while for the wheeled mode, they are 13.75 for the individual policy and 11.01 for the distilled model. Although there is a slight decrease in both cases, no significant performance degradation is observed. In this study, to suppress this performance degradation, a vector that identifies the mode was added as input during distillation to prevent the behaviors of both modes from being completely averaged. However, in principle, it is difficult to suppress this to zero, and it is considered that some of the mode-specific behaviors are still affected by the sharing. Increasing the amount of data during distillation or expanding the network scale is likely to further reduce this difference. On the other hand, considering that without using mode vectors, averaging becomes prominent, and the operation itself may not be feasible, the current results can be evaluated as sufficiently good for practical use. Furthermore, considering the advantages of seamless mode switching, improved inference speed, reduced memory usage, and simplified control systems achieved by single modeling, this degree of performance degradation can be judged to be within an acceptable range.

The primary objective of policy distillation in this research is not to achieve performance maximization with the distilled policy alone, but rather to integrate control strategies for different motion modes into a single representation space and establish an initial state that enables future learning and functional expansion. Therefore, it is expected that performance identical to the specialized policy cannot be achieved immediately after distillation, without additional training. What matters is whether the degree of performance degradation is not excessively large when considering future additional training or feature expansion.

The results shown in Table 4 suggest that, within the scope of the problem setting and evaluation conditions addressed in this study, the performance of the post-distillation policy maintains an order of magnitude comparable to that of the specialized policy. This indicates that it is at least consistent with the possibility of using it as an initial point for additional learning.

Furthermore, Table 5 shows the results of goal arrival times and average travel distances measured on long-distance courses requiring mode switching. When comparing walking mode alone and walking + wheeled mode (both modes at a fixed speed), the average travel distance and time were almost the same, and no increase in the fall rate was observed. Furthermore, in the setting where only the wheeled mode was accelerated, the average travel distance was almost maintained while the time was shortened. In contrast, in the setting where both walking and wheeled modes were accelerated, the average travel distance decreased significantly, and it is presumed that falls increased significantly.

One possible factor is that while both modes can become unstable due to increased speed, the degree of this effect differs between modes. Wheeled mode involves relatively simple motion based on continuous ground contact on flat terrain, making it easier to maintain stability even at higher speeds. In contrast, walking mode is more susceptible to increased destabilizing factors like landing impacts and vertical vibrations during acceleration. Consequently, selectively accelerating only the wheeled mode contributed to reducing overall travel time without significantly increasing major failures.

In summary, while accelerating only the wheeled mode allowed for reduced travel time without compromising stability, accelerating both walking and wheeled modes resulted in decreased stability. Therefore, it was demonstrated that a transformable mobility system that incorporates the wheeled mode with high fall resistance and accelerates only when necessary is effective not only for reducing vibrations but also for improving efficiency in mobility. 

## 7. Discussion

This study excluded transformation actions from the learning target. This is because the transformation process is highly dependent on hardware and involves mechanical elements related to safety, making it difficult to accurately model in simulation. Particularly during transitions between walking and wheeled modes, instability or abnormal contact may occur, potentially causing the robot to fall in real-world scenarios. Therefore, this study focused on analyzing the mobility performance of each mode after transformation under consistent control conditions by forcibly switching modes via an idealized 0/1 transition in simulation. Extensions to control the transformation motion itself more realistically remain a topic for future research.

This study focuses on examining post-transformation motion control in walking mode and wheeled mode, as well as methods for integrating these modes. The transformation process accompanying mode switching and any instability that may arise during this transition are intentionally excluded from evaluation. Consequently, simulations do not reveal any overt falls. However, when considering actual implementation, it is essential to define stability metrics such as fall rate, attitude stability margin, and limits on contact forces and joint torques to quantitatively evaluate safety. The results of this study are positioned as a fundamental investigation to clarify the potential and limitations of mode integration prior to such real-world evaluations.

Furthermore, information such as the joint angles of the chair mechanism and the degrees of freedom of the passenger has not been incorporated into the learning model. While we recognize that this information could potentially influence mobility behavior and stability, it was intentionally excluded in this study. This was done to limit the problem definition and focus on the control characteristics of the mobility modes themselves—walking mode and wheeled mode—and on the methods for integrating them. Including these degrees of freedom from the outset would significantly increase the complexity of the state space and action space, making it difficult to isolate causes of learning instability or performance degradation. Furthermore, accurately simulating the passenger’s active and unpredictable posture changes with high fidelity is currently challenging. Therefore, this study models the passenger as a passive system with mass and inertia, considering its influence solely as load variations and disturbances.

In real-world environments, it is necessary to switch between walking mode and wheeled mode in situations such as just before a step or in narrow passages. Considering the time and energy required for transformation, there may be cases where moving solely in walking mode is more efficient than frequently switching modes. Additionally, from a safety perspective, it is desirable to minimize the number of transformations as much as possible. Therefore, it is anticipated that the proportion of transformation time in the overall movement will be kept to a minimum. However, it is considered necessary to explore methods that balance efficiency and safety by incorporating transformation behavior into the distillation policy through additional learning in the future.

The next consideration is the transferability of simulation results to actual vehicles. In this study, we improved generalization performance through large-scale parallel learning and minimized the discrepancy between simulation and actual vehicles by introducing domain randomization, including passenger models and external disturbances. However, actual vehicle verification has not been conducted, and additional experiments for Sim-to-Real transfer remain a future challenge.

Furthermore, discussions regarding safety are also important. This study assumed the presence of passengers, and in actual operation, “preventing falls” should be the highest priority requirement. In other words, in long-distance environments, even if it takes time, reaching the goal without falling even once is the correct design guideline. To achieve this, it is necessary to incorporate fail-safe mechanisms in addition to strategies. Specifically, auxiliary controls such as emergency stop actions when fall precursors are detected and transition to safety mode when encountering excessive inclines or steps can be considered. Going forward, we believe that the challenge will be to ensure reliability that can withstand real-world operational environments by incorporating such safety measures and integrating them with policy learning.

## 8. Concluding Remarks

Existing wheelchairs and quadruped robots perform well in specific environments, but they have issues with versatility and mobility efficiency. In this study, to overcome these challenges, we verified the effectiveness of a transformable quadruped wheelchair that can switch between walking mode and wheeled mode, as proposed by the authors, in a simulation environment. Specifically, we used reinforcement learning (PPO) to learn walking strategies for uneven terrain and wheel strategies for flat terrain separately and then distilled them into a single integrated policy using mode-conditional policy distillation for evaluation.

The simulation was conducted in an environment built on NVIDIA Isaac Sim, and both modes were trained until the rewards converged using curriculum learning. Post-learning vibration analysis confirmed that the wheeled mode significantly reduces vertical sway compared to the walking mode. Furthermore, by utilizing mode vectors for policy distillation, the two modes were fused into a single policy, demonstrating the potential for improved mobility efficiency in long-distance travel involving mode switching. In particular, it was revealed that selectively increasing speed while leveraging the stability of the wheeled mode could reduce the risk of falling while shortening travel time.

The results of this study demonstrate the effectiveness of an approach that integrates individually learned strategies under mode-dependent conditions in robots with different movement modes. This suggests that this approach could become one of the fundamental technologies enabling efficient and safe traversal in diverse environments. Going forward, promising directions for development include methods for integrating transformational behavior between modes and verification on actual hardware platforms.

## Figures and Tables

**Table 1:** Structure of the Actor Network.

Layer	Features	Activation Function
Input layer	255/259	-
Hidden layer 1	512	ELU
Hidden layer 2	256	ELU
Hidden layer 3	128	ELU
Output Layer	12/16	-

**Table 2:** Structure of the Critic Network.

Layer	Features	Activation Function
Input layer	255/259	-
Hidden layer 1	512	ELU
Hidden layer 2	256	ELU
Hidden layer 3	128	ELU
Output layer	1	-

**Table 3:** Information used as observations.

Item	Description	Number of Dimensions
base_lin_vel	Linear velocity of the robot’s body	(3)
base_ang_vel	Angular velocity of the robot’s body	(3)
projected_gravity	Direction of gravity projected onto the robot’s body frame	(3)
velocity_commands	Velocity commands for movement	(3)
joint_pos	Joint positions of key robot joints	(16)
joint_vel	Joint velocities of key robot joints	(28)
actions	Actions from the previous control step	(12) or (16)
height_scan	Height data scanned from the surrounding terrain	(187)

**Table 4:** Comparison of reward sums for individual policies and integrated policies.

	Individual Policy	Integrated Policy
Walking mode	17.51	16.87
Wheeled mode	13.75	11.01

**Table 5:** Long-distance driving performance comparison results.

	Time Required [s]	Average Travel Distance [m]
Walking mode only	75	53
Walking mode and wheeled mode	75	50
Walking mode and wheeled mode (speed up in wheeled mode only)	62	51
Walking mode and wheeled mode (speed up in both modes)	41	17

## Data Availability

Data Availability Statement: Source code is available at https://github.com/AkamisakaAtsuki/transformable-quadruped-wheelchair-lab (accessed on 24 December 2025).

## Appendix A

**Table A1:** Definitions and physical meanings of each reward term in Equation.

Symbol	Description	Type
$r_{track\_lin\_vel\_xy\_exp}$	Exponential reward for tracking target linear velocity in the XY plane	Positive
$r_{track\_ang\_vel\_z\_exp}$	Exponential reward for tracking target yaw angular velocity	Positive
$r_{lin\_vel\_z\_l2}$	L2 penalty on vertical linear velocity (*Z*-axis)	Penalty
$r_{ang\_vel\_xy\_l2}$	L2 penalty on planar angular velocity (roll/pitch)	Penalty
$r_{joint\_torques\_l2}$	L2 penalty on total joint torque magnitude	Penalty
$r_{joint\_acc\_l2}$	L2 penalty on joint angular acceleration	Penalty
$r_{action\_rate\_l2}$	L2 penalty on the rate of change in actions between consecutive steps	Penalty
$r_{feet\_air\_time}$	Auxiliary reward promoting appropriate foot-air-time during gait cycles (walking mode only)	Auxiliary
$r_{undesired\_contacts}$	Penalty for unintended or untimely ground contacts (walking mode only)	Auxiliary
